# Amyloidosis cutis dyschromica caused by compound heterozygous *GPNMB* mutations in a Chinese pedigree^☆^

**DOI:** 10.1016/j.abd.2024.01.009

**Published:** 2024-10-31

**Authors:** Ci-Juan Zhong, Fang-Gu Li, Wen Li, Yi-Ming Fan

**Affiliations:** a Dermatology, Plastic and Cosmetic Surgery Center, The First Dongguan Affiliated Hospital of Guangdong Medical University, Dongguang, China; b Department of Dermatology, Affiliated Hospital of Guangdong Medical University, Zhanjiang, China

Dear Editor,

Amyloidosis Cutis Dyschromica (ACD, OMIM #617920) is a rare autosomal recessive or dominant disorder caused by Glycoprotein Non-Metastatic b (GPNMB) gene mutation, and characterized by clinically diffuse speckled hyper-/hypopigmentation and pathologically dermal amyloid deposition. To date, 16 different *GPNMB* mutations of 28 ACD pedigrees have been documented in English literature.^1^, ^2^, ^3^, ^4^, ^5^ We describe an autosomal-recessive Chinese ACD family with compound heterozygous *GPNMB* mutations.

A 26-year-old Chinese female presented with a 21-year history of asymptomatic generalized mottled pigmentation. The dyspigmentation began on the limbs and spread progressively to the whole body. Several pruritic blisters occurred recurrently on the arms during summertime and healed spontaneously without scarring, but photosensitivity was absent. Cutaneous examination showed numerous reticulate hyper-/hypopigmented macules involving almost the entire body, with mild involvement of the face and neck and sparing of the dorsal of hands and feet (Fig. 1A). Hair, nails, teeth, and mucosae were normal. Two siblings had similar lesions (Fig. 1B-C), but other members including non-consanguineous parents were not affected. Dermoscopy displayed ill-defined, irregular, white macules surrounded by brownish pigmentation, and indistinct linear vessels (Fig. 1D). Laboratory examinations including full blood count, urinalysis, biochemical and antinuclear antibody profile, chest X-Ray, and abdominal ultrasonography were unremarkable. Light microscopy from an arm lesion revealed: a hyperkeratotic and partially atrophic epidermis with basal layer hypopigmentation in hypopigmented area; and hyperkeratotic epidermis with mild hyperpigmentation and focal basal liquefaction degeneration, amorphous eosinophilic deposits and sparse melanophages in the papillary dermis in hyperpigmented area (Fig. 2A). Masson-Fontana stain showed hypermelanosis in hyperpigmented area. The deposits stained positive with Congo red stain and high-molecular-weight cytokeratin CK34βE12 immunostaining (Fig. 2B). Additional skin biopsies were obtained from other members (II2, II4 and II5). Congo red stain and CK5/6 immunostaining displayed abundant amyloid deposits in hyperpigmented lesions and little in hypopigmented lesions in three affected subjects, and absent in an II4 carrier with c.565C > T. Immunohistochemically, cytoplasmic GPNMB expression in the basal and suprabasal layers was weak in hyperpigmented lesions or absent in hypopigmented lesion of ACD patients, and moderate in forearm skin of II4 carrier with c.565C > T and normal control (Fig. 2C-F). Electron microscopy disclosed intracytoplasmic fibrillar aggregates in degenerated basal keratinocytes and partial destruction of basal lamina and cytomembrane between the uppermost amyloid deposits and the basal cells in some areas. Homogeneous fibrillar bodies were surrounded by collagen bundles and fibroblastic and histiocytic processes in the papillary dermis (Fig. 3).

**Figure 1:**
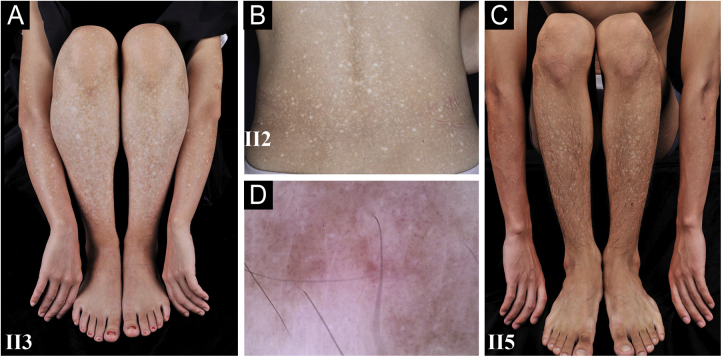
Clinical and dermoscopic observation. (A‒C) Diffuse hyperpigmentation intermingled with numerous hypopigmented macules on the limbs and back in 3 ACD patients, without the involvement of the dorsa of hands and feet. (D) Dermoscopy displayed ill-defined, irregular, white macules surrounded by brownish pigmentation, and indistinct linear vessels (original magnification ×60).

**Figure 2:**
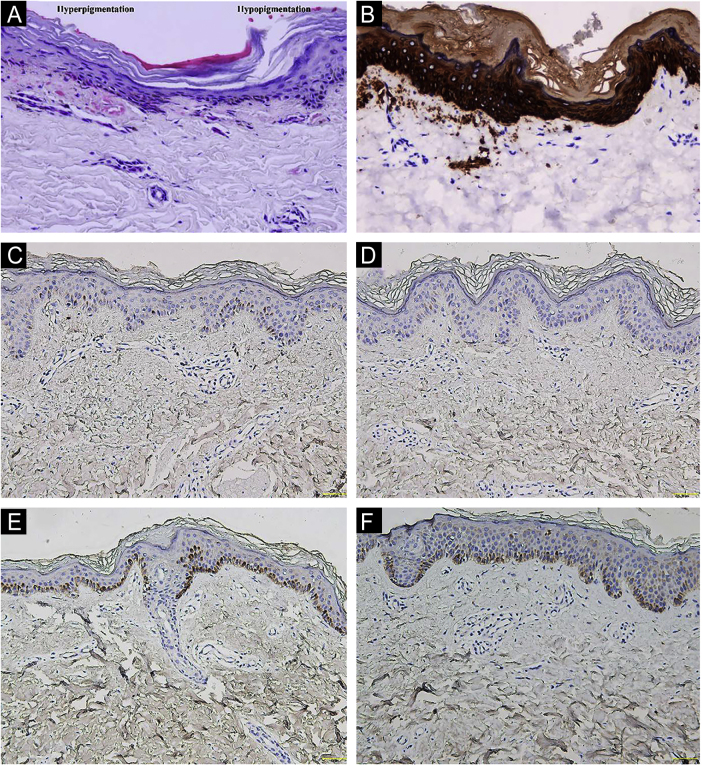
Histopathological and immunohistochemical observation. (A) Hematoxylin-eosin stain showed hyperkeratotic epidermis with mild hyperpigmentation and focal basal liquefaction degeneration, amorphous eosinophilic deposits and sparse melanophagesin the papillary dermis (left side); and hyperkeratotic and partially atrophic epidermis with basal layer hypopigmentation (right side) (original magnification ×200). (B) Cytokeratin 34βE12 immunostaining revealed amyloid deposits in the upper dermis (original magnification×200). (C‒F) GPNMB immunoreactivity was weak in hyperpigmented lesion (C) and absent in hypopigmented lesion (D) of II5 patient, and moderate in forearm skin of II4 carrier with c.565C>T (E) and normal control (F) (original magnification ×200).

**Figure 3:**
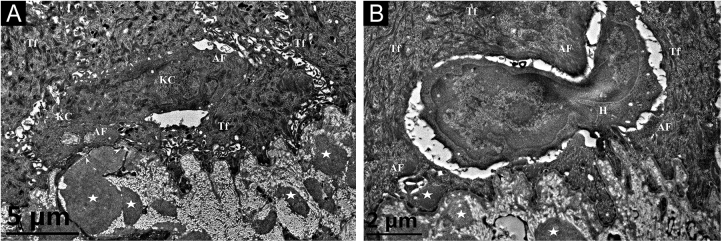
Transmission electron microscopy. (A) Cytoplasmic Amyloid-like Filaments (AF) in 2 degenerated basal Keratinocytes (KC), Tonofilaments (Tf) in adjacent normal keratinocytes, partial disruption (arrowheads) of basal lamina and cytomembrane between the uppermost amyloid deposit and the degenerated basal cell, and fibrillar bodies (asterisks) in the papillary dermis (original magnification x8,000). (B) A Histiocyte (H) lay between 2 partly degenerated basal keratinocytes containing AF and Tf, partial disruption (arrowhead) of basal lamina and cytomembrane, and fibrillar bodies (asterisks) in the papillary dermis (original magnification x15,000).

Whole exome and Sanger sequencing of peripheral blood DNA identified compound heterozygous mutations of c.565C > T (p.R189*) in exon 5 and c.1092delT (p.P365Lfs*21) in exon 7 of *GPNMB* in 3 affected siblings (Fig. 4A-B), which were respectively derived from her mother and father. Three affected siblings were diagnosed as ACD, and skin lesions of the proband remained stable at a 4.5-year follow-up.

**Figure 4:**
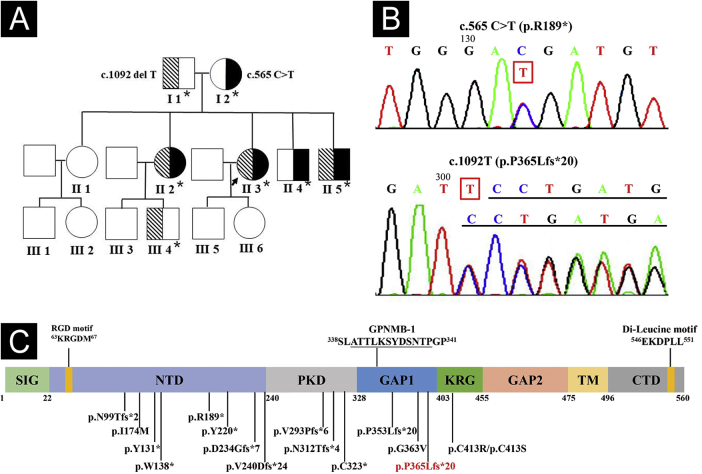
Pedigree and GPNMB sequencing and protein structure. (A) Pedigree of the family. Asterisks represent the participants with genetic testing. (B) Sanger sequencing of the proband showed compound heterozygous mutations of c.565C>T in exon 5 and c.1092delT in exon 7 of *GPNMB*. (C) Structure and 16 mutations of human GPNMB protein originating from 17 *GPNMB* mutants (p.V240Dfs*24 deriving from mutants c.717_718delTG and c.719_720delTG). A novel c.1092delT mutation in this pedigree is marked in red. Mutants GPNMB-1 (a splice isoform of GPNMB) with an in-frame 12-amino acid insertion (underlined), and RGD and Di-Leucine motifs are also shown. SIG, Signal Sequence Domain; NTD, N-Terminal Domain; PKD, Polycystic Kidney Disease-like domain; KRG, Kringle-like domain; TM, Transmembrane Domain; CTD, C-Terminal Cytoplasmic Domain.

Compound heterozygous *GPNMB* mutations of a known c.565C > T and a novel c.1092delT were found in this family. Hence, 17 ACD-associated *GPNMB* mutations of 29 ACD pedigrees (including our case) have been identified, namely, 7 (41.2%) frameshift, 5 (29.4%) nonsense, 4 (23.5%) missense, and 1 (5.9%) splice site mutations (Fig. 4C).^1^, ^2^, ^3^, ^4^, ^5^ Mutant c.565C > T is a common nonsense mutation in the East Asian population and a founder mutation in Chinese ACD patients, resulting in premature termination (p.R189*) in the N-terminal domain of GPNMB.^1^, ^2^ Furthermore, similar to mutant c.1056delT (p.P353Lfs*20),^1^ c.1092delT(p.P365Lfs*21) may be a novel frameshift mutation causing premature termination between polycystic kidney disease-like and Kringle-like domains of GPNMB.

GPNMB is highly expressed in melanocytes and pivotal for melanosome formation.^1^ GPNMB expression was decreased in hypopigmented lesions of ACD and vitiligo patients.^2^ The heterozygous *GPNMB* carriers with c.700 + 5G > T presented with mild hyperpigmentation and GPNMB downexpression and no amyloid deposition in a semi-dominant pedigree.^3^ However, a carrier with c.565C > T manifested as normal phenotype and GPNMB expression in our pedigree with recessive ACD. These results suggest *GPNMB* haploinsufficiency cannot contribute to ACD phenotype.^2^

The amyloid deposits were abundant in hyperpigmented lesions and little in hypopigmented lesions, and positive for CK5/6 and CK34βE12.^1^, ^4^ Electron microscopy revealed homogeneous fibrillar bodies in the papillary dermis and intracytoplasmic fibrillar aggregates in degenerated keratinocytes.^1^, ^3^ Conditioned media from GPNMB-silenced melanocytes increased keratinocyte apoptosis.^3^ These results indicate that degenerative and necrotic keratinocytes could contribute to amyloid formation.^1^ Although ACD-associated mutations could cause aberrant GPNMB localization,^2^ its role in ACD pathomechanism remains to be further elucidated.

## Financial support

This study was supported by the Discipline Construction Project of Guangdong Medical University (4SG21277P).

## Authors’ contributions

Ci-Juan Zhong: Study conception and planning; data collection, analysis and interpretation; manuscript preparation and writing.

Fang-Gu Li: Study conception and planning; data collection, analysis and interpretation; manuscript preparation and writing.

Wen Li: Data collection, analysis and interpretation.

Yi-Ming Fan: Study conception and planning; data collection, analysis and interpretation; manuscript critical review and final version approval.

## Conflicts of interest

None declared.
